# Adipose browning response to burn trauma is impaired with aging

**DOI:** 10.1172/jci.insight.143451

**Published:** 2021-08-23

**Authors:** Abdikarim Abdullahi, Carly M. Knuth, Christopher Auger, Thibacg Sivayoganathan, Alexandra Parousis, Marc G. Jeschke

**Affiliations:** 1 Faculty of Medicine, University of Toronto, Toronto, Ontario, Canada.; 2 Biological Sciences, Sunnybrook Research Institute, Toronto, Ontario, Canada.; 3 Ross Tilley Burn Centre, Sunnybrook Hospital, Toronto, Ontario, Canada.; 4 Department of Surgery, Division of Plastic Surgery, and Department of Immunology, University of Toronto, Toronto, Ontario, Canada.

**Keywords:** Aging, Endocrinology, Adipose tissue, Uncoupling proteins

## Abstract

**BACKGROUND:**

The incidence of burn injuries in older patients is dramatically increasing as the population of older people grows. Despite the increased demand for elderly burn care, the mechanisms that mediate increased morbidity and mortality in older trauma patients are unknown. We recently showed that a burn injury invokes white adipose tissue browning that leads to a substantially increased hypermetabolic response associated with poor outcomes. Therefore, the aim of this study was to determine the effect of age on the metabolic adipose response of browning after a burn injury.

**METHOD:**

One hundred and seventy patients with burn injury admitted to the Ross Tilley Burn Centre were prospectively enrolled and grouped by age as older (≥50 years) and young (≤35 years). Adipose tissue and sera were collected and analyzed for browning markers and metabolic state via histology, gene expression, and resting energy expenditure assays.

**RESULTS:**

We found that older patients with burn injury lacked the adipose browning response, as they showed significant reductions in uncoupling protein 1 (UCP1) expression. This failure of the browning response was associated with reduced whole-body metabolism and decreased survival in older patients with burn injury. Mechanistically, we found that the adipose of both aged patients after burn trauma and aged mice after a burn showed impairments in macrophage infiltration and IL-6, key immunological regulators of the browning process after a severe trauma.

**CONCLUSION:**

Targeting pathways that activate the browning response represents a potential therapeutic approach to improve outcomes after burn trauma for elderly patients.

**FUNDING:**

NIH (R01-GM087285-01), Canadian Institutes of Health Research (grant no. 123336), and Canada Foundation for Innovation Leaders Opportunity Fund (no. 25407).

## Graphical abstract

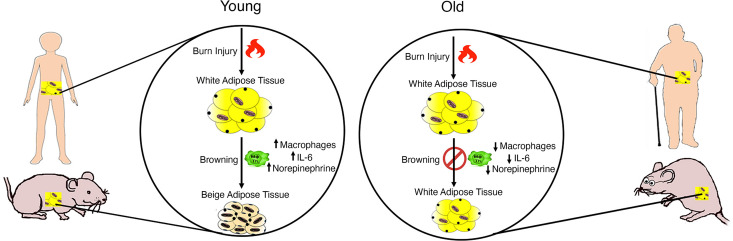

## Introduction

According to the World Health Organization, the elderly are the fastest growing segment of the population; this population is projected to increase to 2 billion people by 2050. It is also expected that people over 65 years of age will represent almost 39% of trauma admissions across North America by 2050 (1). This will constitute a major healthcare problem worldwide and specifically in developed countries that boast higher life expectancies. Indeed, studies have shown that older patients after burn trauma (hereafter referred to as older burn patients) have a higher risk of death and severe disability after trauma, despite having similar comorbidities and severity scores to their younger counterparts (2–4). Many believe that the increase in morbidity and mortality that is seen in older trauma patients is partially due to the substantial decline of cellular function that leads to their impaired capacity to respond to severe injuries. For instance, impaired adaptive and homeostatic mechanisms in key metabolic and immune organs in elderly people result in diminished physiological reserves and an inability to meet the heightened demands of the traumatic injury (5, 6). In fact, studies in older burn patients have implicated a weakened immunity and an inability to fight infections after burn injury, along with immunosenescence and altered skin morphology and thinning leading to deeper burns (7–9). Despite the recognized impairments in immunity and skin physiology in mediating poor outcomes of older trauma patients, the metabolic alterations with aging and their role in after trauma outcomes in these patients remains unclear.

Perhaps the most serious gap in our understanding of the age-associated metabolic decline of trauma patients is the role of the adipose tissue, in particular how adipose tissue remodeling from white- to brown-like adipocytes affects energy expenditure and outcomes (10–12). Whereas white adipose tissue (WAT) is responsible for storing large amounts of triglycerides, brown adipose tissue (BAT) is characterized by smaller lipid droplets, increased mitochondrial biogenesis, and the expression of uncoupling protein 1 (UCP1), which uncouples ATP generation from oxidative phosphorylation, thus dissipating energy as heat (13–15). Unfortunately, both BAT activity and quantity have been shown to decline during aging, with diminished reserves occurring as early as the mid-40s in humans and in midlife for rodents (11). Recently, a more physiologically significant type of brown-like adipocyte that also expresses UCP1, termed beige/brite adipocytes, have been identified within WAT depots in response to β adrenergic activation under cold conditions (16). Disease and pathological conditions, such as cancer and burn trauma, have also recently been reported to induce beige adipocyte formation and, subsequently, body weight and fat loss (17–20). Although the age-dependent decline in BAT is well characterized, whether a similar age-dependent decline in the formation of injury-induced beige adipocytes is unknown. Deciphering the age-associated changes in beige fat activation, particularly during nonphysiological states, such as burn trauma, will help us to devise new therapies that could potentially reverse the age-associated impairments in fat oxidation and fuel availability that mediate poor outcomes during the injury (21).

The aim of the following study was to examine the alterations to the browning process with aging during trauma. We report impaired beige adipocyte formation and increased mortality in both an aged murine model of thermal injury and clinical burn patients. Impairments in burn-induced browning were not a delayed response, as aged mice failed to exhibit beige adipocyte formation even up to 1 month after injury. Impaired macrophage recruitment to the WAT of both murine animals and clinical patients mediated the failure in browning in response to the burn trauma. Specifically, the two chief regulators of burn-induced browning, IL-6 and alternatively activated macrophages, were both decreased in aged mice. Thus, our findings support the notion that an age-dependent failure to form beige adipocytes is not only present in cold-induced physiological states, but also exists during conditions of injury.

## Results

### Failure of burn-induced beige adipocyte formation in older patients.

**Figure 1:**
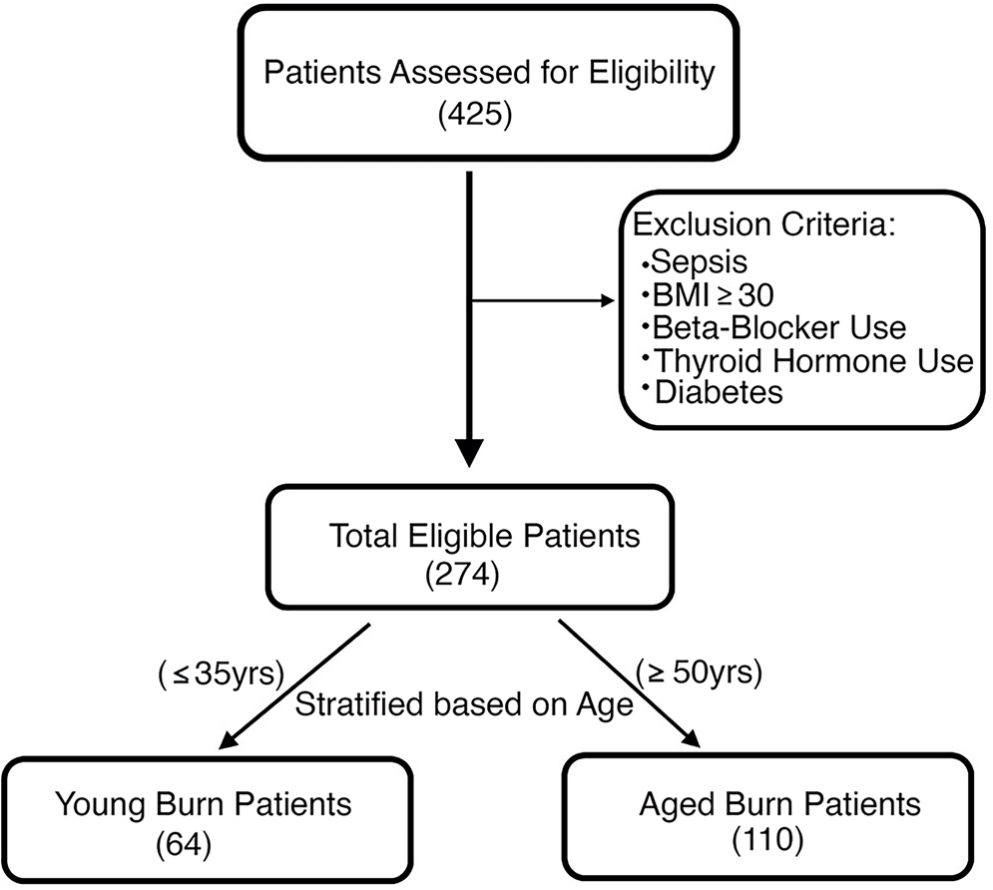
CONSORT flow diagram showing enrollment of patients and allocation.

To assess whether aging impairs the WAT browning response observed after burn trauma, we retrospectively enrolled 425 burn patients admitted to our trauma burn center (Figure 1). After assessing for eligibility, we enrolled 64 young, severely burned patients (26 ± 6 years old) with burns encompassing 37% ± 16% of their total body surface area (TBSA) (Table 1). We also recruited 110 older severely burned patients (63 ± 10 years old) with burns covering 34% ± 14% of their TBSA (Table 1). Young control patients without burn injury (30 ± 4 years old) and older patients (57 ± 8 years old) were also recruited into our study (Table 2). Subcutaneous WAT (sWAT) samples collected from enrolled patients were stratified into two groups based on age.

**Figure 2:**
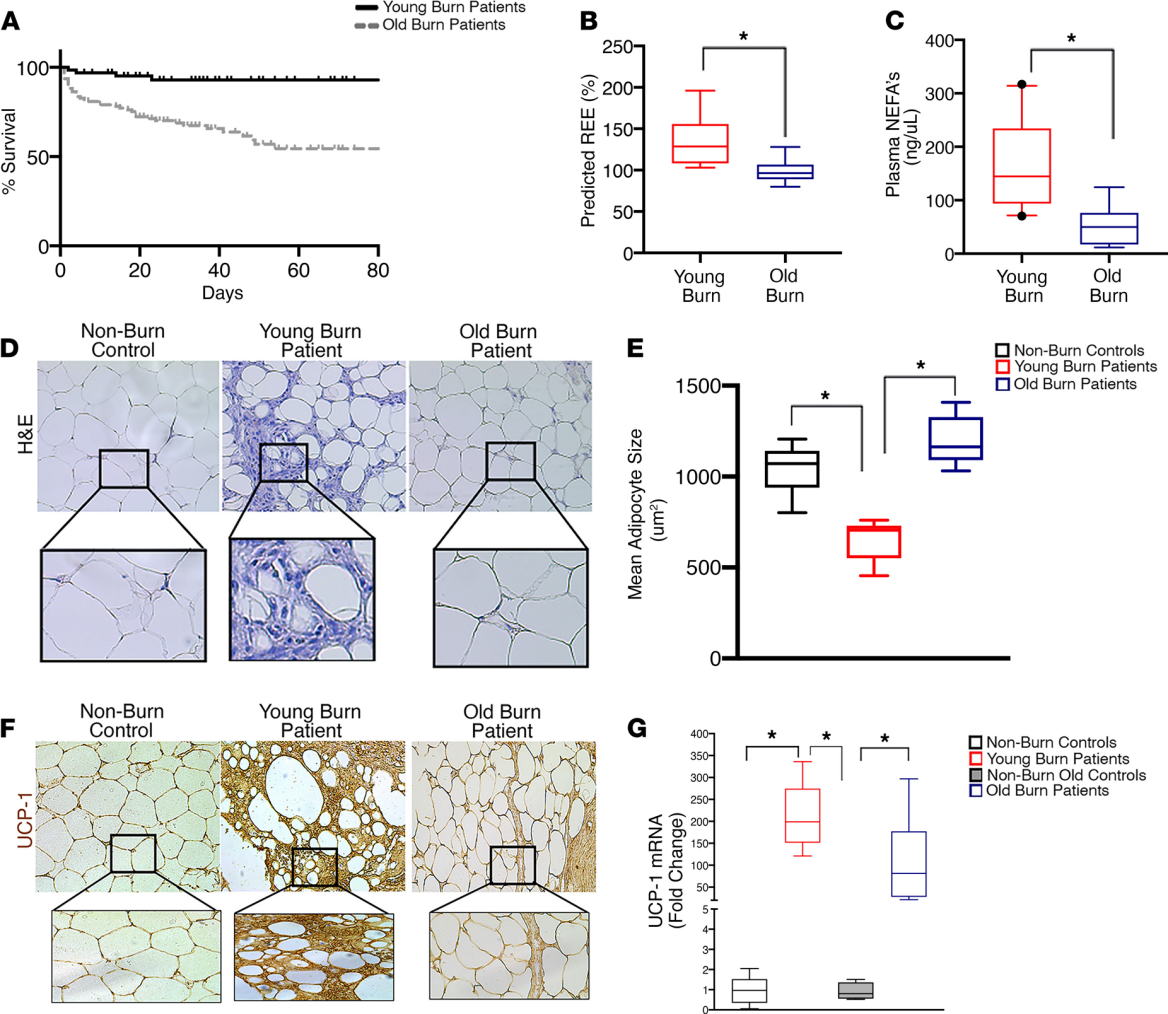
Failure of burn-induced beige fat formation in aged burn patients. (**A**) Kaplan-Meier survival curves of young and older burn patients with burns over 20% of total body surface area. (**B**) Box-and-whisker plots showing average measured and predicted REE in young and older burn patients. (**C**) Box-and-whisker plots showing plasma concentration of free fatty acids in young and older burn patients. (**D**) Immunohistochemistry H&E staining of subcutaneous WAT of young and older burn patients. (**E**) Quantification of average lipid droplet size in subcutaneous WAT isolated from young and older burn patients. (**F**) Immunohistochemistry staining of UCP1 in subcutaneous WAT of young and older burn patients. (**G**) Box-and-whisker plots showing quantitative RT-PCR analysis of browning gene UCP1 in subcutaneous WAT isolated from young and older burn patients. The box plots depict the 5%–95% quartiles (whiskers), the upper and lower quartiles, and the median. The length of the box represents the interquartile range. Data represent the mean ± SEM. *P < 0.05, young vs. older burn patients (*n* = 55–107). Scale bars: 100 μm. Statistical differences were determined using an unpaired *t* test with Welch’s correction.

We found that older burn patients (29%) had a significantly higher overall mortality rate compared with that of younger burn patients (7%) (Figure 2A). Whole-body resting energy expenditure (REE) and lipolysis measurements also revealed that older burn patients had a reduced metabolic rate after injury compared with that of younger burn patients (Figure 2, B and C). This was further associated with a decline in adipose remodeling and beige adipocyte formation in older burn patients (Figure 2, D and E). Compared with sWAT from control patients without burn injury, sWAT from young burn patients stained positively for UCP1 and contained multilocular cells (Figure 2, D–F). In contrast to young patients, sWAT from older patients showed no evidence of browning following burn injury, as assessed by the lack of staining for UCP1 and the absence of multilocular cells (Figure 2, D–F). In corroboration with our immunohistochemistry data, UCP1 mRNA expression was higher in the sWAT of young burn patients relative to that in the older burn group (Figure 2G). When the data were further stratified into young (≤35 years) and elderly (≥65 years) groups, the effects were more pronounced within the elderly subgroup, showing a greater increase in mortality and a more pronounced decrease in adipose browning (Supplemental Figure 1; supplemental material available online with this article; https://doi.org/10.1172/jci.insight.143451DS1). Together, these findings suggest that beige adipocyte formation after a burn trauma may have therapeutic relevance and the loss of browning in older burn patients may be associated with poor outcomes.

### Failure of burn-induced beige adipocyte formation in aged mice.

**Figure 3:**
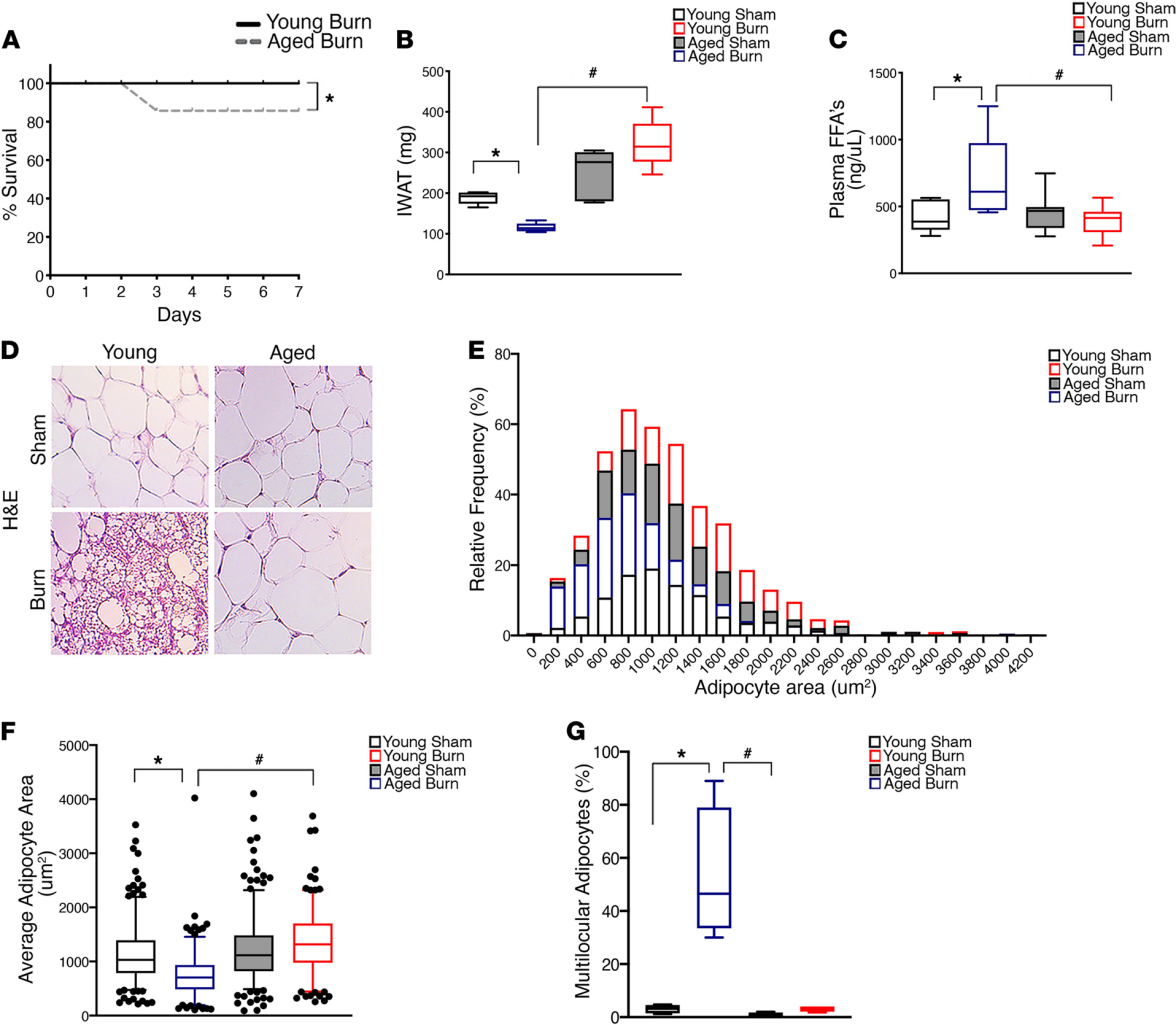
Increased mortality and impaired adipose remodeling in aged mice after a burn injury. (**A**) Kaplan-Meier survival curves of young vs. aged mice subjected to a 30% total body surface area burn injury. (**B**) Changes in inguinal WAT mass in young and aged mice 1 week after burn injury. (**C**) Plasma concentration of free fatty acids in young and aged mice 1 week after burn injury. (**D**) Immunohistochemistry H&E staining of subcutaneous WAT of young and aged mice. (**E**) Different lipid droplet size frequency in subcutaneous WAT isolated from young and aged mice. (**F**) Quantification of average lipid droplet size in subcutaneous WAT isolated from young and aged mice. (**G**) Quantification of the number of multilocular adipocytes in subcutaneous WAT isolated from young and aged mice. Data represent the mean ± SEM. *P < 0.05, vs. sham; ^#^*P* < 0.05, vs. burn (*n* = 10). Scale bars: 100 μm. Statistical differences were determined using 2-way ANOVA followed by Bonferroni post hoc test.

**Figure 4:**
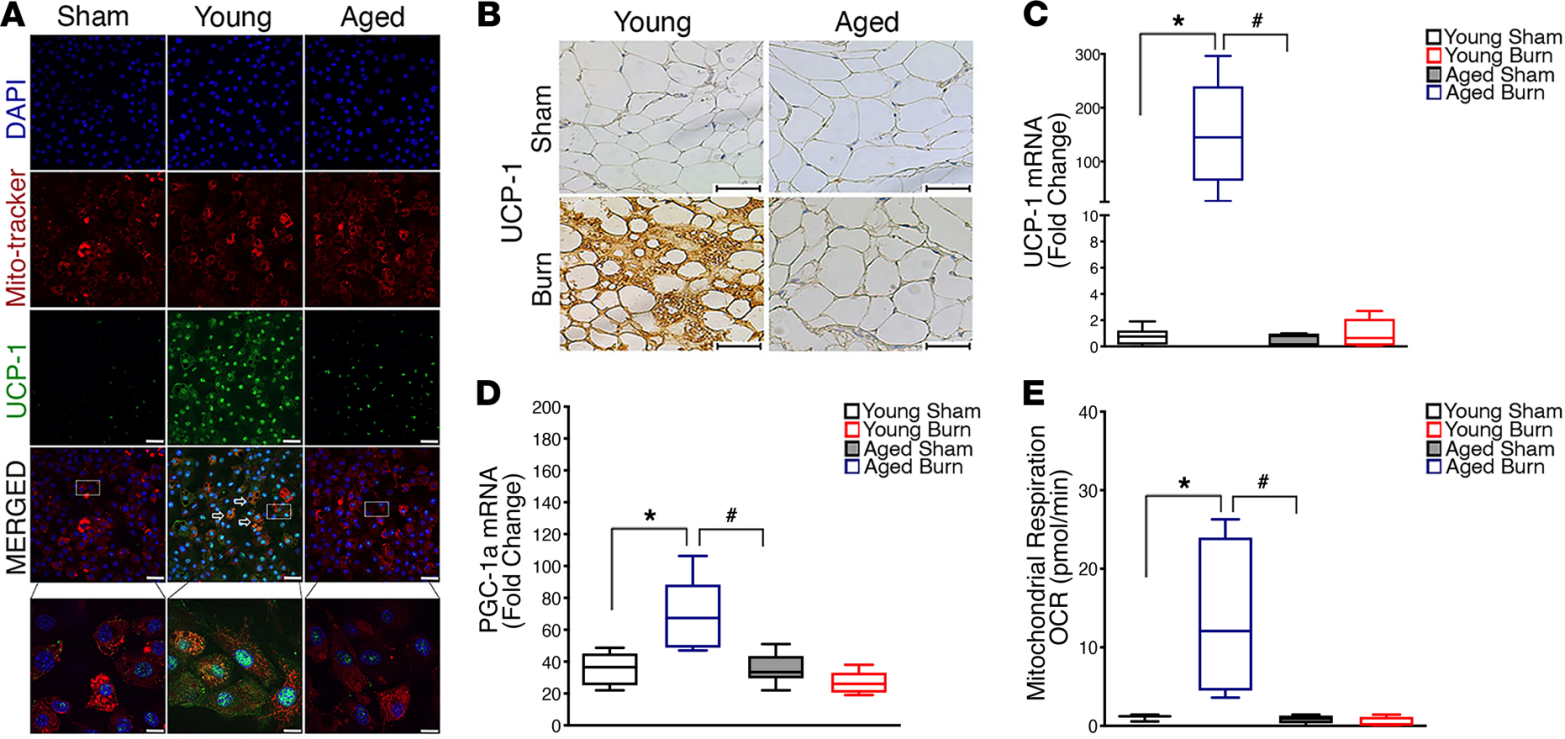
Failure of burn-induced beige fat formation in aged mice. (**A**) Differentiated isolated adipocytes were fixed and then stained for MitoTracker (Red), UCP1 (Green), and nuclei (DAPI) and then imaged (original magnification, ×60). (**B**) Immunohistochemistry staining of UCP1 in inguinal WAT of young and aged mice 1 week after burn injury. (**C**) Quantitative RT-PCR analysis of browning gene UCP1 in inguinal WAT of young and aged mice 1 week after burn injury. (**D**) Quantitative RT-PCR analysis of mitochondrial biogenesis gene PGC1α in inguinal WAT of young and aged mice 1 week after burn injury. (**E**) Analysis of mitochondrial oxygen consumption rate in isolated inguinal WAT of young and aged mice 1 week after burn injury. Data represent the mean ± SEM. *P < 0.05, vs. sham; ^#^*P* < 0.05, vs. burn (*n* = 6–9). Scale bars: 100 μm. Statistical differences were determined using 2-way ANOVA followed by Bonferroni post hoc test.

To examine if the observations from our clinical elderly burn patients could be recapitulated in a murine model, we next examined whether trauma-induced browning was also impaired in aged mice. To do this, we utilized a well-characterized animal burn injury model whereby C57BL/6J mice that were either 8 weeks old (young mice) or 52 weeks old (aged mice) and were then randomized into either the control or 30% TBSA burn injury group (Supplemental Figure 2) (22). Similar to the increased mortality we observed in elderly burn patients, aged mice subjected to a severe burn injury also showed an increase in mortality relative to their younger counterparts (Figure 3A). This increased mortality in aged mice after burn injury was also associated with a reduced metabolic response, as assessed by the lack of tissue wasting and fat metabolism in these mice (Figure 3, B and C). Consistent with adipose remodeling after burn injury and increased fat metabolism, analysis of sWAT from young mice showed reduced lipid droplet size and increased multilocular adipocytes compared with those in aged mice (Figure 3, D–G). Furthermore, young mice exposed to a burn trauma formed beige adipocytes and expressed the key browning marker UCP1 within the inguinal sWAT depot (Figure 4, A–C). Conversely, aged mice exposed to a burn trauma failed to form beige adipocytes and exhibited a blunted induction of the browning gene, UCP1 (Figure 4, A–C). Additionally, the chief regulator of mitochondrial biogenesis peroxisome proliferator–activated receptor γ coactivator 1 α (PGC1A) and mitochondrial respiration were also increased in young mice and blunted in aged mice after burn injury, further confirming an impairment in browning in aged mice (Figure 4, D and E). We further assessed whether the impaired browning response to burn trauma in aged mice persisted throughout the course of the injury past the 1 week time point. Indeed, while young mice 2 weeks after burn trauma still showed signs of browning, such as UCP1^+^ beige adipocytes and increased mitochondrial respiration, these responses failed to develop in the aged mice at this time point (Supplemental Figure 3, A–C). This blunted adipose browning response observed in aged mice persisted even after 1 month after burn trauma compared with that in their younger counterparts (Supplemental Figure 4, A–C). Thus, these findings further advance the notion that aging impairs the adipose browning response to trauma and that aged mice fail to recover this potentially therapeutic metabolic process, even as the injury manifests over time.

### Key regulators of burn-induced browning are diminished in both aged mice and older burn patients.

**Figure 5:**
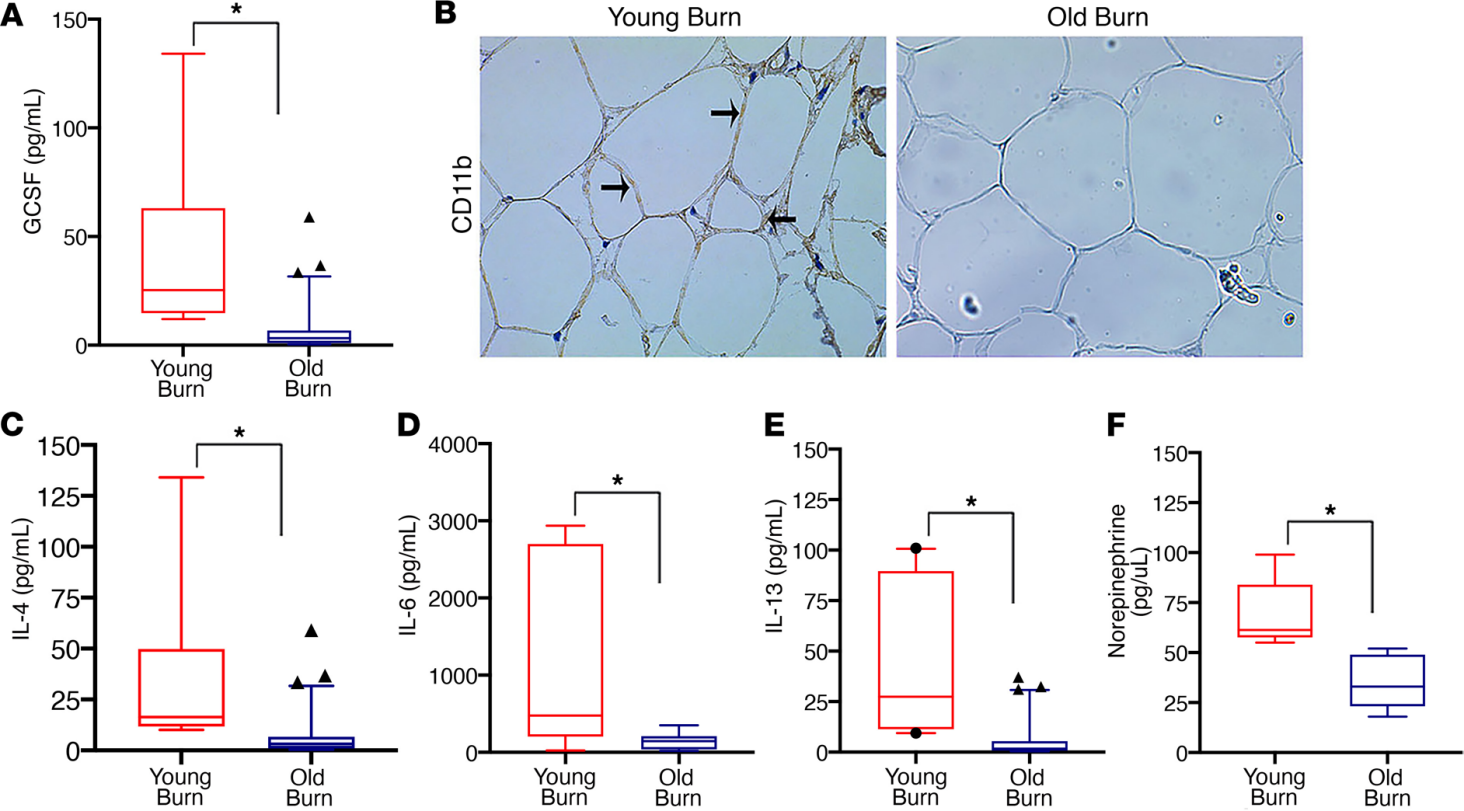
Blunted macrophage recruitment and decreased expression of cytokines involved in the regulation of burn-induced browning in older patients. (**A**) Box-and-whisker plots showing expression levels of the key protein GCSF involved in the mobilization of macrophages in the plasma of young and older burn patients. (**B**) Immunohistochemistry staining of macrophage marker CD11b in subcutaneous WAT isolated from young and older burn patients. Arrows indicate CD11b^+^ cells. (**C**) Box-and-whisker plots showing plasma concentration of IL-4 in young and older burn patients. (**D**) Box-and-whisker plots showing plasma concentration of IL-6 in young and older burn patients. (**E**) Box-and-whisker plots showing plasma concentration of IL-13 in young and older burn patients. (**F**) Norepinephrine content in sWAT isolated from young and older burn patients. The box plots depict the 5%–95% quartiles (whiskers), the upper and lower quartiles, and the median. The length of the box represents the interquartile range. Data represent the mean ± SEM. *P < 0.05, young vs. older burn patients (*n* = 15–30). Scale bars: 50 μm. Statistical differences were determined using an unpaired *t* test with Welch’s correction.****

**Figure 6:**
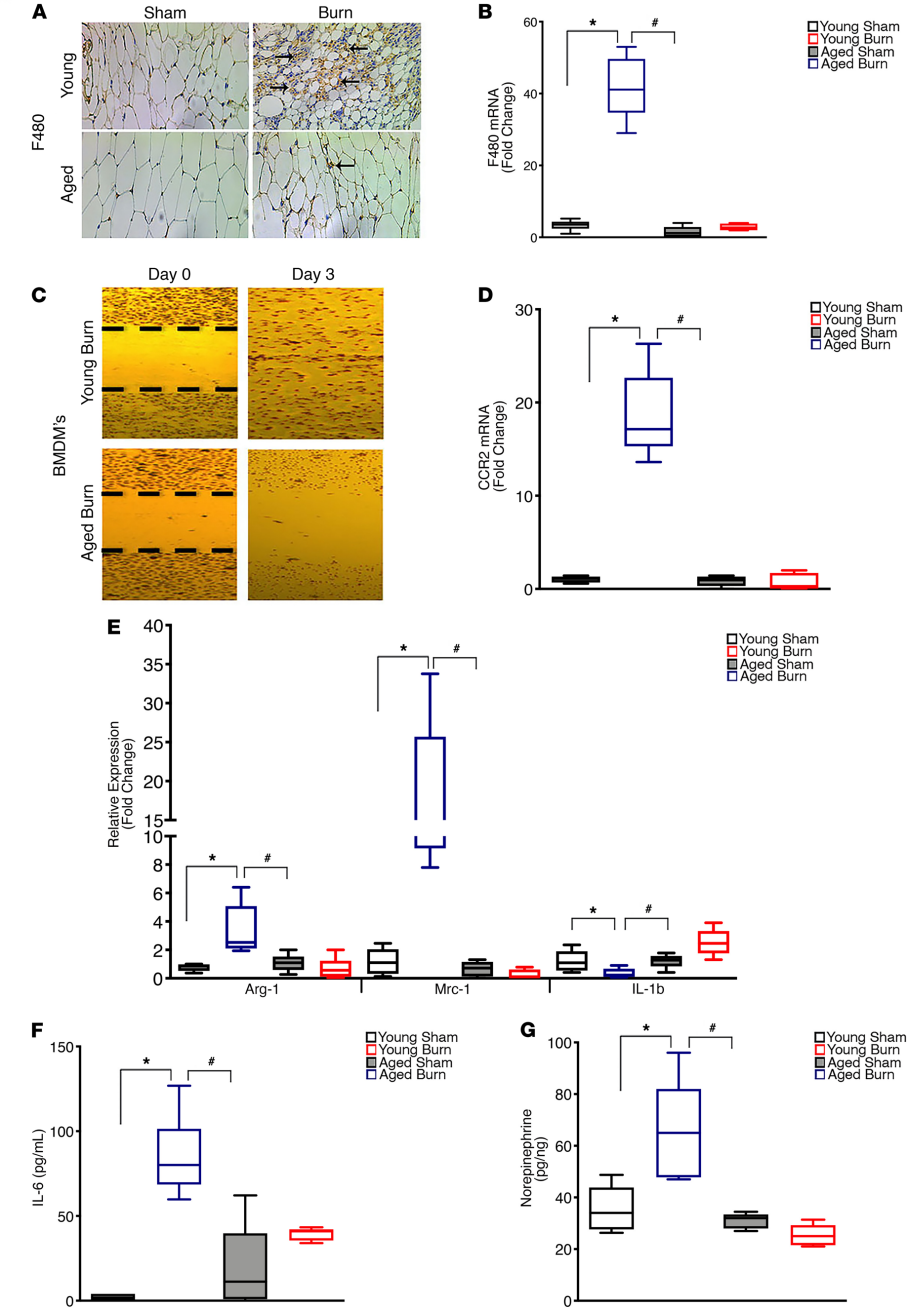
Aging impairs macrophage chemotaxis and the secretion of cytokines involved in the regulation of burn-induced browning in mice. (**A**) Immunohistochemistry staining of macrophage marker F4/80 in inguinal WAT of young and aged mice 1 week after burn injury. (**B**) Quantitative RT-PCR analysis of macrophage marker F4/80 in inguinal WAT of young and aged mice 1 week after burn injury. (**C**) In vitro chemotaxis assay of bone marrow–derived macrophages isolated from young and aged mice 1 week after burn injury. (**D**) Quantitative RT-PCR analysis of macrophage chemotaxis marker CCR2 in inguinal WAT of young and aged mice 1 week after burn injury. (**E**) Quantitative RT-PCR analysis of M2 macrophage markers in inguinal WAT of young and aged mice 1 week after burn injury. (**F**) Plasma concentration of IL-6 in young and aged mice 1 week after burn injury. (**G**) Norepinephrine content in inguinal WAT of young and aged mice 1 week after burn injury. Data represent the mean ± SEM. *P < 0.05, vs. sham; ^#^*P* < 0.05, vs. burn (*n* = 10). Scale bars: 100 μm. Statistical differences were determined using 2-way ANOVA followed by Bonferroni post hoc test.****

Recently, the cytokines IL-6, IL-4 and alternatively activated macrophages that secrete norepinephrine have been implicated in mediating beige adipocyte formation during burn trauma, cancer, calorie restriction, and cold-induced WAT browning (18, 23–27). As such, we hypothesized that aging also interferes with the expression of the aforementioned key regulators required to form beige adipocytes in response to burn trauma. To test this hypothesis in humans, we first assessed the recruitment of macrophages to the adipose tissue, a critical first step in the adipose browning process, as we have previously shown (23). We found both a decrease in granulocyte colony-stimulating factor (GCSF), the key chemokine involved in macrophage chemotaxis, and the number of infiltrated CD11b^+^ macrophages in the sWAT of older burn patients compared with that in their younger counterparts (Figure 5, A and B). Additionally, older burn patients showed decreased plasma levels of the key cytokines (IL-4, IL-6, IL-13) and norepinephrine, which have been shown to regulate WAT browning in burns (23, 24) (Figure 5, C–F). Similarly, in response to burn injury, aged mice showed an impaired macrophage migratory capability, which was associated with a decrease in the number of infiltrated F4/80^+^ macrophages within sWAT (Figure 6, A–D). Not only did the aged mice show impairments in macrophage migration and recruitment, but they also showed decreases in circulating IL-6 levels, alternatively activated macrophages, and norepinephrine, which are all requirements for the activation of the browning process during a burn trauma (23, 24) (Figure 6, E–G). All together, these findings suggest that the disruption of the adipose browning response to trauma with aging is, at least in part, mediated by reduced macrophage recruitment, deficiencies in key cytokines, and the subsequent norepinephrine secretion required to initiate the browning process.

## Discussion

Metabolic adaptation occurs after a severe trauma in order to better optimize nutrient usage. This metabolic response that includes adipose browning is critical to the hypermetabolic response that is activated after a burn trauma in order to drive host survival. Several lines of evidence have demonstrated that the dysregulation of whole-body energy expenditure and substrate oxidation can have dire effects on the metabolic health and disease outcomes of elderly patients (28–30). Specifically, changes in adipose tissue physiology and function that control whole-body energy homeostasis have been implicated in mediating altered metabolism with aging (5, 8, 11). Older patients after burn trauma are at a significantly increased risk for complications and death as a result of degenerative changes in metabolism (e.g., dwindling energy expenditure, mitochondrial function, accumulating adiposity, and substantial decline in muscle mass) (3, 8, 31). The present work has uncovered some important features of WAT remodeling in response to trauma during aging. Here, we found an age-related impairment in the formation of beige adipocytes after injury in patients and aged mice, which was associated with increased mortality following a burn trauma. We further found a failure to activate adipose browning in response to burn trauma in aged mice at as early as 1 week; this impairment remained even after 1 month after injury. Interestingly, we found a decrease in key contributors to beige adipocyte formation in response to trauma, including diminished macrophage adipose infiltration and a parallel decrease in IL-6 in aged mice and older patients. Thus, our data suggest that therapeutically restoring adipose browning could potentially improve both the metabolic state and overall outcome in aged burn trauma patients.

Previous studies have predominantly focused on the aging effects on BAT, leaving the age-related changes in beige adipocyte formation largely unexplored. Indeed, only two other studies have identified age-related changes in browning, with both studies yielding similar results to ours presented here (32, 33). For instance, it has been recently reported that cold-induced beige adipocyte formation is impaired both in aged mice and patients (32). Similarly, another recent study found that female mice have a progressive age-dependent loss of sWAT browning, and this process runs in parallel with morphological changes in BAT (33). Our studies differ in that we are the first to our knowledge to assess the effects of aging on WAT browning in the context of an injury and not in a physiological context, such as cold exposure. These variations between our studies and those previously reported are important as the physiological and cellular activities activated during cold exposure and in trauma or cancer, where browning has been reported, are starkly different (16, 18, 23). Furthermore, while we have previously reported that chronic browning in adult burn patients fuels persistent hepatic steatosis and hypermetabolism, this should not suggest that all beige activity is bad per se (34, 35). We believe that a certain amount of beige activity may be beneficial in burns by helping to mobilize energy reserves to establish adequate wound healing and protective immune responses. Indeed, our findings herein, in which reduced beige adipocyte activity with aging was associated with poorer outcomes after a burn injury, are not contradictory to our previous findings. Rather, our studies suggest that activation of WAT browning in either extreme (too little vs. too much) are problematic for patient outcomes. Ultimately, further research is required to enable more nuanced monitoring of this browning process in burns during aging so that we can better identify the optimal activation levels of this remarkable process for its therapeutic potential.

Complementary studies that have utilized cold exposure models to induce browning have leant credence to the notion that age-dependent beige progenitor dysfunction might, in part, be attributed to cellular senescence (32). For example, genetic and pharmacologic manipulation of p38/MAPK and the Ink4a/Arf pathway, 2 key senescence regulators, was sufficient to block cold-inducible beige adipocyte formation in young mice (36). Conversely, genetic and pharmacological inhibition of the Ink4a/Arf pathway blunted senescence in older animals and restored metabolic benefits of cold-induced browning (32, 37). However, the age-related changes to the key regulators of WAT browning outside of senescence have not been studied. This is particularly important, as most of the literature on browning has implicated both macrophages and the cytokines IL-4 and IL-6 in the formation of beige adipocytes (23–27). Our potentially novel findings in rodents and clinical burn patients have demonstrated impairments in macrophage migration and IL-6 production, necessary factors for trauma-induced browning. This suggests that the age-dependent failure in browning is not only due to senescence of adipocytes, but also changes in the regulators of browning as well. Thus, pharmacologic or other approaches that could modulate macrophage activity and IL-4/IL-6 production in the adipose tissue during aging could be exploited therapeutically in the recovery of beige fat to potentially improve metabolic health, as been done in the field of obesity (38, 39).

In summary, our findings indicate that aging results in a profoundly reduced adipose browning response to burn trauma and that this is associated with significantly worse outcomes. Subsequent questions to be investigated include whether these findings are also true in other hypermetabolic conditions, such as cancer, where adipose browning has also been reported. Further understanding of the regulation and alterations of the WAT browning metabolic response with aging in conditions outside of cold exposure will provide new directions and possibly targeted therapies to improve the metabolic health status of older trauma patients.

## Methods

### Human samples.

We retrospectively enrolled 425 burn patients admitted to our trauma burn center. After assessing for eligibility based on our exclusion criteria (sepsis, BMI ≥ 30, beta blocker use, thyroid hormone use, and diabetes), those patients that remained were stratified into two age groups; young burn patients (26 ± 6 yr) and older burn patients (63 ± 10 yr) (Supplemental Figure 1). The age cutoff chosen for the older burn patient cohort (>50 yr) was selected based on previous studies that reported a significant decline in WAT browning and brown fat after this age (29, 32, 33). sWAT was collected from the torso or lower/upper limbs of all patients enrolled, depending on the type of burn injury sustained. Adipose tissue was only collected from patients who had surgeries that occurred ≥10 days after their injury, as previous studies have shown browning is only evoked after this time period (19, 20). Tissue samples collected were immediately transferred to the laboratory and either frozen in liquid nitrogen (–80**°**C) or placed in fixative for histology until analysis. REE was measured with a Sensor Medics 2900 metabolic measurement cart.

### Animals.

Male C57BL/6J mice that were 6 weeks or 1 year old were purchased from The Jackson Laboratory (stock no. 000664) and housed at ambient temperatures of approximately 23°C–24°C. Mice were cared for in accordance with the *Guide for the Care and Use of Laboratory Animals* (National Academies Press, 2011). After arrival, mice were acclimated to our facility for 2 weeks before the commencement of the experiments. By the date of experiments, the mice were either 8 weeks old (young mice) or 52 weeks old (aged mice).

### Mouse thermal injury model.

We used a previously well-characterized rodent burn injury model (22). Mice received a 30% TBSA full-thickness burn. All mice were anesthetized with 2.5% isoflurane and shaved along the dorsal spine region. Ringer’s lactate (2–3 mL) was injected subcutaneously in all treatment mice to protect the spine, and buprenorphine (0.05–0.1 mg/kg body weight) was injected before and after the burn injury when necessary, as required for pain management. A full-thickness third-degree dorsal scald burn encompassing 15% TBSA and a 15% wound on the ventral surface was induced by immersing the dorsum of the mice in 98°C water for 10 seconds and the ventral region for 2 seconds. Mice were subsequently housed individually in sterile cages and fed ad libitum until sacrifice. Sham (control) mice underwent identical experimental procedures, with the exception of the burn injury. WAT (inguinal, epididymal) and plasma were collected upon sacrifice and stored in –80°C until analysis.

### Histology and immunohistochemistry.

Adipose tissues were immediately fixed in 10% formalin and then maintained in 70% ethanol before paraffin embedding. Subsequently, tissues were sectioned and stained with H&E or incubated with UCP1 (U6382, MilliporeSigma), F4/80 (MF48000AbD, Thermo Fisher), or CD11c (ab52632, Abcam) antibody followed by DAB staining. Imaging was performed on a LSM confocal microscope (Zeiss).

### Quantitative PCR.

Total RNA isolated from adipose tissues was analyzed by quantitative RT-PCR. Briefly, RNA was isolated from tissues using TRIzol-chloroform (Life Technologies) with subsequent purification using the RNeasy Kit (Qiagen) according to the manufacturer’s instructions. RNA (2 mg) was transcribed to cDNA using the high-capacity cDNA reverse transcription kit (Applied Biosystems). Real-time quantitative PCR was performed using the Applied Biosystems Step One Plus Real-Time PCR System. Primer sequences are available in Supplemental Table 1.

### Primary macrophage culture and conditioned medium preparation.

Mice were sacrificed by cervical dislocation and were rinsed in 70% (vol/vol) ethanol. Then, bone marrow was isolated from femurs and tibias. Bone marrow cells were plated at a density of 1 × 10^6^ to 2 × 10^6^ cells/mL in RPMI-1640 medium (supplemented with 10% FCS, 1% glutamine, 1% penicillin-streptomycin, and 10 ng/mL macrophage colony-stimulating factor [PeproTech]) on 6-well plates and were allowed to differentiate for 7 days. After differentiation, these BMDMs were used for an in vitro scratch assay.

### Cytokine profile.

Rodent and human sera were collected, and a panel of cytokines was measured using a Luminex platform (MilliporeSigma).

### Primary adipocyte culture.

Isolated mouse SV cells were cultured in Dulbecco’s modified Eagle medium with 10% FBS (Invitrogen) at 37°C in a 5% CO_2_ environment. Beige adipogenesis was induced by treating confluent cells with DMEM containing 10% FBS, 0.5 mM isobutylmethylxanthine, 125 nM indomethacin, 1 mM dexamethasone, 850 nM insulin, 2 nM T3, and 1 mM rosiglitazone (Alexis Biochemicals). Differentiated adipocytes were then stained with MitoTracker Red (Thermo Fisher), UCP1 (U6382, MilliporeSigma), and DAPI (BD Biosciences).

### Respiration assays.

Oxygen consumption rates were measured in freshly excised adipose tissues (inguinal adipose depots). Tissues were minced in mitochondrial isolation buffer (MHSE + BSA; 210 mM mannitol, 70 mM sucrose, 5 mM HEPES, 1 mM EGTA, 0.5% [w/v] fatty acid–free BSA, pH 7.2). The tissue was then homogenized using a Teflon glass homogenizer. Mitochondria were isolated via differential centrifugation. Briefly, the homogenate was centrifuged at 600*g* for 10 minutes, and the supernatant decanted into a new tube. This fraction was centrifuged at 9000*g* for 10 minutes to obtain a mitochondrial pellet, which was subsequently resuspended in 50 μL MHSE + BSA. BCA assays (Thermo Scientific) were performed to gauge protein concentrations. Mitochondrial bioenergetics were assessed using a Seahorse XF96 analyzer (Agilent Technologies). Mitochondrial respiration in a coupled state (10 μg/well) was measured in mitochondrial assay solution (220 mM mannitol, 70 mM sucrose, 10 mM KH_2_PO_4_, 5 mM MgCl_2_, 2 mM HEPES, 1 mM EGTA, and 0.2% [w/v] fatty acid–free BSA, pH 7.2, at 37°C) containing succinate as a substrate (10 mM) and rotenone (2 μM). State 3 respiration (phosphorylating respiration) was triggered via the injection of a cocktail containing 4 mM ADP along with 10 mM pyruvate, 2.5 mM glutamate, and 2.5 mM malate. State 4o respiration was assessed by the addition of 2.5 μg/mL oligomycin, while maximal uncoupler-stimulated respiration was observed following the injection of 4 μM carbonyl cyanide 4-(trifluoromethoxy)phenylhydrazone (FCCP). Antimycin A (4 μM), a complex III inhibitor, was added at the end of the experiment to inhibit mitochondrial respiration. The Seahorse XF Wave software was used to analyze the data.

### Lipolysis assay.

For in vivo lipolysis assays in mice, serum samples were collected, and free fatty acid levels were then quantified per the manufacturer’s instructions (Biovision Abcam). Serum fatty acids from patients were analyzed by gas chromatography–mass spectrometry performed by the Analytical Facility for Bioactive Molecules platform at the Hospital for Sick Children (Toronto, Ontario, Canada). Briefly, serum samples (20 μL) were spiked with an internal standard mix and acidified with HCl. Nonesterified fatty acids were acidified and double extracted with hexane. The fatty acids were then converted to their pentafluorobenzyl esters using 1% pentafluorobenzyl bromide/diisopropylamine (1:1) and separated by automated gas chromatography (GC Agilent 7890A, Agilent Technologies) on a fused-silica SP2380 capillary column (30 m × 0.25 mm × 0.2 μm film thickness; Supelco Analytical). Fatty acid ions were detected and measured using a MSD Agilent 5975C quadrupole mass detector (Agilent Technologies). Peaks of fatty acid esters were identified by comparisons with individual fatty acid standards (Supelco Analytical).

### Catecholamine assay.

Norepinephrine was quantified using an Elisa kit (BAE-5200R, Rocky Mountain Diagnostics) per the manufacturer’s protocols. For norepinephrine ELISAs, tissues were homogenized by sonication in homogenization buffer (0.01 N HCl, 1 mM EDTA, 4 mM Na_2_S_2_O_5_), and cellular debris was pelleted by centrifugation at 15,115*g* for 15 minutes at 4°C. The cleared homogenates were collected and stored in a –80°C freezer before quantification. All samples were normalized to total tissue protein concentration.

### Statistics.

All data are presented as mean ± SEM and were analyzed using Prism 9 (GraphPad). Normality and equal variance were verified in all data. Data were log transformed if normal distribution and/or equal variance tests failed. Statistical differences between 2 groups were evaluated using 2-tailed unpaired *t* test with Welch’s correction, where appropriate, for single variables. Statistical differences among 3 or more groups were evaluated using 2-way ANOVA followed by Bonferroni post hoc test. Results were considered significant at *P* < 0.05.

### Study approval.

Patients admitted to the Ross Tilley Burn Centre at Sunnybrook Health Sciences Centre (Toronto, Ontario, Canada) and patients without burn undergoing elective surgery preoperatively provided consent for tissue and blood collection. Approval for our human study was obtained from the Research Ethics Board at Sunnybrook Health Sciences Centre. All animal studies and procedures were approved by the Sunnybrook Research Institute Animal Care Committee under AUP 467.

## Author contributions

AA performed the experiments, analyzed the data, and wrote the manuscript. CMK and CA performed the experiments, analyzed the data, and edited the manuscript. TS and AP performed the experiments. MGJ edited the manuscript and conceptualized the paper.

## Supplementary Material

Supplemental data

Trial reporting checklists

ICMJE disclosure forms

## Figures and Tables

**Table 1:**
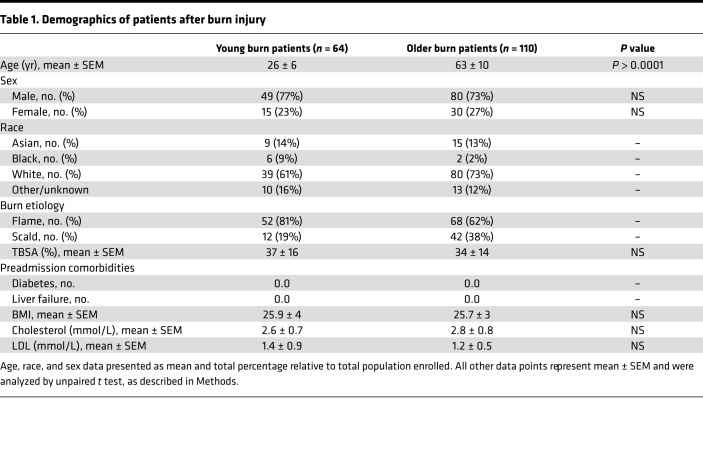
Demographics of patients after burn injury

**Table 2:**
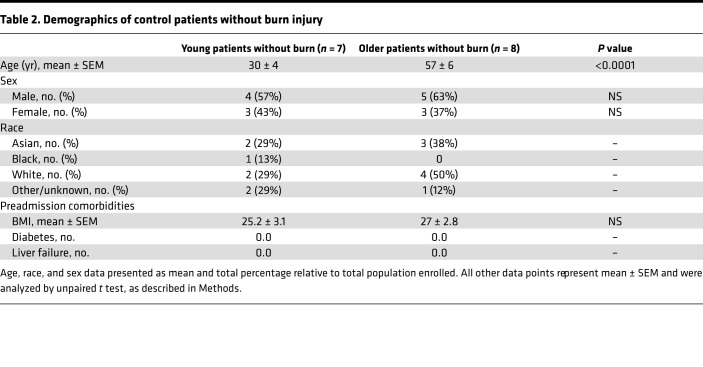
Demographics of control patients without burn injury

## Version 1 08/23/2021

Electronic publication
