# Supplementary material for: Adipose browning response to burn trauma is impaired with aging
Source: JCI Insight. 2021 Aug 23;6(16):e143451. doi: 10.1172/jci.insight.143451 (PMC8409980; doi:10.1172/jci.insight.143451)
Supplement: Supplemental data [file jciinsight-6-143451-s009.pdf]

Supplemental Data Set:

Supplemental Table 1: Primer Sequences.

Supplemental Figure 1. Failure of burn-induced beige fat formation in Elderly burn patients. (**A**) Kaplan Meier survival curves of young (<35yrs) vs. elderly burn patients (>65yrs) with burns over 20% total body surface area (TBSA). (**B**) Box-and-whisker plots showing average measured predicted resting energy expenditure (REE) in young (<35yrs) vs. elderly burn patients (>65yrs). (**C**) Immunohistochemistry staining of UCP1 in subcutaneous WAT of young (<35yrs) vs. elderly burn patients (>65yrs). (**D**) Box-and-whisker plots showing quantitative RT-PCR analysis of browning gene UCP1 in subcutaneous WAT isolated from young (<35yrs) vs. elderly burn patients (>65yrs). (**E**) Box-and-whisker plots showing expression levels of the key protein GCSF involved in the mobilization of macrophages in the plasma of young (<35yrs) vs. elderly burn patients (>65yrs). (**F**) Box-and-whisker plots showing plasma concentration of IL6 in young (<35yrs) vs. elderly burn patients (>65yrs). (**G**) Box-and-whisker plots showing plasma concentration of IL4 in young (<35yrs) vs. elderly burn patients (>65yrs). The box plots depict the 5-95% quartiles (whiskers), the upper and lower quartiles, and the median. The length of the box represents the interquartile range. Data represented as mean ± SEM, $p<0.05$ * = significant difference Young vs Old Burn Patients. Statistical differences was determined using an Unpaired t-test with Welch's correction.

Supplemental Figure 2. Schematic Illustration of the animal burn injury model.

Supplemental Figure 3. Impaired burn-induced beige fat formation in aged mice two weeks post-injury. (**A**) H and E and immunohistochemistry staining of UCP1 in inguinal WAT of burned young and aged mice two weeks post-injury. (**B**) Quantitative RT-PCR analysis of browning gene UCP1 in inguinal WAT of young and aged mice two weeks post-injury. (**C**) Analysis of mitochondrial oxygen consumption rate in isolated inguinal WAT of young and aged mice two weeks post-injury. Data represented as mean ± SEM, $p<0.05$ * = significant difference vs. sham, $p<0.05$ # = significant difference vs. burn, (n=6). Statistical differences were determined using two-way ANOVA followed by Bonferroni post-hoc test.

Supplemental Figure 4. Persistent failure in beige adipocyte formation in aged mice post-burn injury. (**A**) Immunohistochemistry staining of UCP1 in inguinal WAT of burned young and aged mice one-month post-injury. (**B**) Quantitative RT-PCR analysis of browning gene UCP1in inguinal WAT of young and aged mice one-month post-injury. (**C**) Analysis of mitochondrial oxygen consumption rate in isolated inguinal WAT of young and aged mice one-month post-injury. Data represented as mean ± SEM, $p<0.05$ * = significant difference vs. sham, $p<0.05$ # = significant difference vs. burn, (n=6). Statistical differences were determined using two-way ANOVA followed by Bonferroni post-hoc test.

Supplemental Table 1: Primer Sequences

NAME	SPECIES	FORWARD	REVERSE
GAPDH	MOUSE	5'-AAGGTGAAGGTCGGAGTCAAC-'3	5'-GGGGTCATTGATGGCAACAATA-'3
ARG-1	MOUSE	5'-AGACCACAGTCTGGCAGTTG-'3	5'-CCACCCAAATGACACATAGG-'3
CCR2	MOUSE	5'-AGGAGCCATACCTGTAAATGC-'3	5'-TGTGGTGAATCCAATGCCCT-'3
IL1B	MOUSE	5'-ATGATGGCTTATTACAGTGGCAA-'3	5'-GTCGGAGATTCGTAGCTGGA-'3
Mrc-1	MOUSE	5'-TGATTACGAGCAGTGGAAGC-'3	5'-GTTCACCGTAAGCCCAATTT-'3
UCP1	MOUSE	5'-CACCTTCCCGCTGGACACT-'3	5'-CCCTAGGACACCTTTATACCTAATGG-'3
PGC-1A	MOUSE	5'-TTCCACCAAGAGCAAGTAT-'3	5'-CGCTGTCCCATGAGGTATT-'3
18s	HUMAN	5'-GGGAGGTAGTGACGAAAAAT-'3	5'-ACCAACAAAATAGAACCGCG-'3
UCP1	HUMAN	5'-AGGTCCAAGGTGAATGCCC-'3	5'-TTACCACAGCGGTGATTGTTC-'3

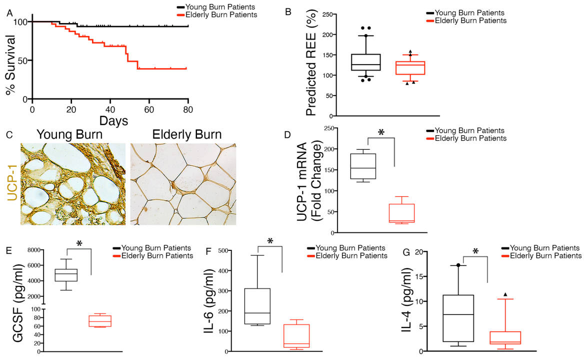
A
Young Burn Patients
Elderly Burn Patients
% Survival
Days
B
Predicted REE (%)
C
Young Burn
Elderly Burn
UCP-1
D
UCP-1 mRNA (Fold Change)
E
GCSF (pg/ml)
F
IL-6 (pg/ml)
G
IL-4 (pg/ml)

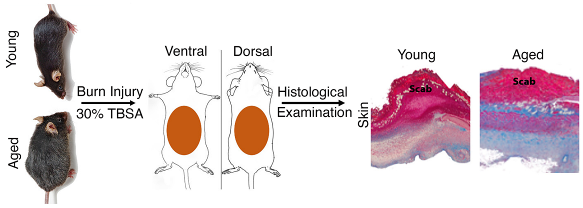
Young
Aged
Burn Injury
30% TBSA
Ventral
Dorsal
Histological
Examination
Skin
Young
Aged
Scab
Scab

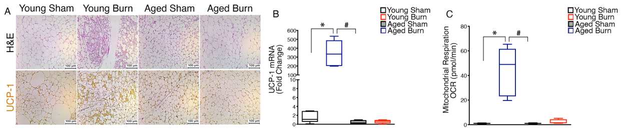
A
Young Sham
Young Burn
Aged Sham
Aged Burn
H&E
UCP-1
100 µm
B
UCP-1 mRNA (Fold Change)
Young Sham
Young Burn
Aged Sham
Aged Burn
C
Mitochondrial Respiration OCR (pmol/min)
Young Sham
Young Burn
Aged Sham
Aged Burn

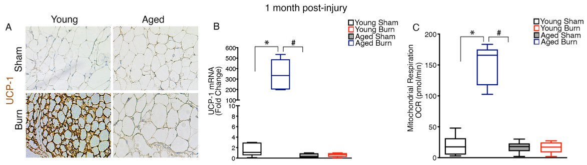
1 month post-injury
A
Young
Aged
Sham
Burn
UCP-1
B
UCP-1 mRNA (Fold Change)
Young Sham
Young Burn
Aged Sham
Aged Burn
C
Mitochondrial Respiration OCR (pmol/min)
Young Sham
Young Burn
Aged Sham
Aged Burn
