# Supplementary material for: Adipose browning response to burn trauma is impaired with aging
Source: JCI Insight. 2021 Aug 23;6(16):e143451. doi: 10.1172/jci.insight.143451 (PMC8409980; doi:10.1172/jci.insight.143451)
Supplement: Trial reporting checklists [file jciinsight-6-143451-s010.pdf]

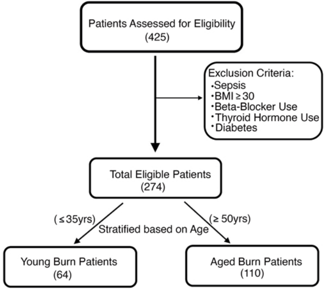
Patients Assessed for Eligibility
(425)
Exclusion Criteria:
•Sepsis
•BMI ≥ 30
•Beta-Blocker Use
•Thyroid Hormone Use
•Diabetes
Total Eligible Patients
(274)
(≤ 35yrs)
(≥ 50yrs)
Stratified based on Age
Young Burn Patients
(64)
Aged Burn Patients
(110)
